# Applying population mechanistic modelling to find determinants of chimeric antigen receptor T-cells dynamics in month-one lymphoma patients

**DOI:** 10.1093/immadv/ltaf001

**Published:** 2025-06-09

**Authors:** Liam V Brown, Mark McConnell, Robert Rosler, Leanne Peiser, Brian J Schmidt, Alexander V Ratushny, Eamonn A Gaffney, Mark C Coles

**Affiliations:** Wolfson Centre for Mathematical Biology, Mathematical Institute, University of Oxford, United Kingdom; Kennedy Institute of Rheumatology, University of Oxford, United Kingdom; AstraZeneca, Cambridge, United Kingdom; Chinook Therapeutics, Seattle, WA, United States; Bristol Myers Squibb, Seattle, WA, United States; Bristol Myers Squibb, Seattle, WA, United States; Bristol Myers Squibb, Seattle, WA, United States; Bristol Myers Squibb, Princeton, NJ, United States; Bristol Myers Squibb, Seattle, WA, United States; Wolfson Centre for Mathematical Biology, Mathematical Institute, University of Oxford, United Kingdom; Kennedy Institute of Rheumatology, University of Oxford, United Kingdom

**Keywords:** chimeric antigen receptor, simulation, modelling, clinical trials, lymphodepletion

## Abstract

**Background:**

Chimeric antigen receptor (CAR) T-cells have been utilized for the treatment of several malignancies, including Non-Hodgkin lymphomas. A myriad of product- and patient-specific factors determines the extent of patient response, and determining which are most impactful requires analysis of clinical data.

**Methods:**

We used population-level ordinary differential equation models to fit clinical flow cytometry and tumour biopsy data from the TRANSCEND-NHL-001 (NCT02631044) study [1]. We analyzed the impact of lymphodepletion, CAR T-cell phenotypes, and other factors on CAR T-cell dynamics for 30 days after infusion.

**Results:**

We quantified the relative contribution of antigen-dependent and independent sources of proliferation on CAR T-cell dynamics, finding that both make a large contribution and that antigen-independent proliferation was highly correlated with patient IL-15 and IL-7 blood concentrations. The proportion of CAR T-cells in naïve, memory, or effector cells was found to have a limited impact on CAR T-cell dynamics, compared with lymphodepletion and tumour burden.

**Conclusions:**

This study shows how models can be used to link endogenous T-cells, CAR T-cells, and their phenotypes, and may be useful for determining whether a given patient may be responding poorly to treatment, by observing the dynamics of their endogenous T-cells. The framework we developed can be utilized for other CAR T constructs and indications, to test product alterations or biological hypotheses at the population level.

## Graphical Abstract

**Graphical Abstract:**
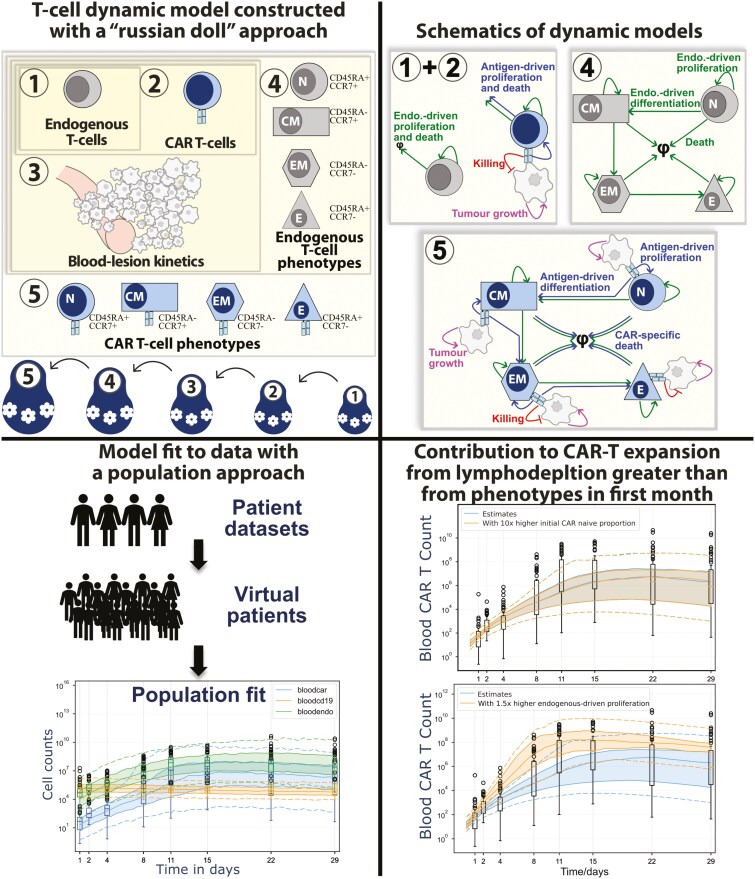
Top-left: hierarchical structure used to construct the model of CAR T-cell dynamics. Top-right: schematic of dynamic models. Model 3 is not shown, which simply adds blood-lesion interchange. Bottom-left: a virtual population approach was used to fit data (box plots are data, shaded regions and dashed lines are quartiles and 90 percentiles of model outputs). Bottom-right: CAR T-cell expansion in the first month after infusion is predicted to be strongly influenced by dynamics that impact endogenous and CAR T-cells equally (‘endogenous-driven proliferation’, linked to lymphodepletion), whereas a substantial change to initial phenotype proportions is predicted not to have as much impact. These results are explored in Section 3.3. N: Naïve T-cells, CM: Central memory, EM: Effector memory, E: Terminal effector. Endo.: Endogenous.

## Introduction

Chimeric antigen receptor (CAR) T-cells are a promising modality for oncological applications. They are T-cells that have been harvested from a patient and transduced with a CAR that recognizes a tumour-associated antigen. They have seen clinical success in many indications, particularly haematological disorders, but many challenges still need to be addressed, including targeting to tumour sites and reduction of on-target/off-tumour effects, reduction of toxicity (especially neurological) and cytokine release syndrome, and limitations on their efficacy such as poor expansion, immune cell regulation, exhaustion, or anergization. Alongside clinical trials, there have been many mathematical models of CAR T-cell dynamics. Stein *et al.* produced a phenomenological model of tisagenlecleucel which describes the early expansion followed by biphasic decline [2]. Singh *et al.* produced a model that could describe the kinetics and dynamics of various CAR T-cells *in vitro* and in mouse models [3], by combining a physiologically based pharmacokinetic model to a model of CAR-T-tumour dynamics, to reproduce the multiphasic profile identified by Stein *et al.* and to evaluate a dose–response relationship. They later extended the model to a translational clinical study [4].

Many factors contribute to CAR T-cell efficacy. The dose–response relationship is relatively weak, since transferred CAR T-cells will immediately begin to proliferate, changing the effective dose to an extent that depends on patient-specific factors: cellular proliferation propensity, a conducive cytokine environment, the extent of proliferation the patient’s body can support, and tumour (antigen) burden—too little, and CAR T-cell proliferation may not be stimulated; too much, and the eventual effector/target ratio may be insufficient for tumour clearance. Patients typically undergo lymphodepletion in preparation for receiving CAR T-cells, as this has been found to promote cellular expansion. Long-term response is known to correlate with the relative amounts of naïve or memory cells [5], exhausted cells [5], or regulatory cells [6, 7]. Which factors have the greatest impact, in which patients (who), at what times (when) and through which mechanisms (why), are still not quantitatively understood. In this study, we seek to determine the factors that are the strongest determinants of patient response (or lack thereof) in the first 30 days after transfusion of anti-CD19 CAR T-cells. Lisocabtagene maraleucel (liso-cel) is an approved, autologous, CD19-directed, 4-1BB CAR T-cell product. The phase 1 study, TRANSCEND NHL-001 [1], recruited 344 patients with PET-positive relapsed or refractory diffuse large B-cell lymphoma, high-grade lymphoma with rearrangements in MYC and either BCL2, BCL6, or both, primary mediastinal B-cell lymphoma, or follicular lymphoma grade 3B. Of these, 256 received liso-cel and could be evaluated for efficacy. Of those, we considered 64 that have a high cross-section of data usable for model fitting. We constructed a series of mechanistic models that predict the numbers of endogenous T-cells (defined as cells not introduced with the CAR product), CAR^+^ T-cells, and CD19^+^ target (B-) cells in the blood and tumour lesions. We encoded proliferation, death and killing of cells, naïve, central memory, effector memory and effector phenotypes, and lymphodepletion into the model.

**Study aim:** to find which biological features are the strongest determinant of CAR T-cell dynamics and patient response, and how changes to the product or other controllable factors might be expected to affect dynamics and response in the first 30 days after transfusion, by fitting the models to the population of patient data.

## Materials and Methods

### Summary of models

We created models to evaluate the observable impact of endogenous and CAR T-cells, T-cell phenotypes, and kinetics and dynamics. We chose to implement four phenotypes: naïve, central memory, effector memory, and terminal effector which differentiate linearly in that order. These four states were chosen to match available data, and an irreversible progression was chosen rather than a branching structure as this was a better fit to observations.

The models are defined in a ‘Russian doll’ formation, where each additional layer of the model extends the previous layers without affecting them. For example, processes that impact endogenous T-cells, such as lymphodepletion-induced proliferation, are also assumed to affect CAR T-cell dynamics, but CAR T-cell dynamics such as antigen stimulation are assumed not to affect endogenous T-cells. In total, we utilized five model layers:

(i) Endogenous T-cells (i.e. T-cells not introduced with the CAR product) in the blood
(ii) Endogenous and CAR T-cells in the blood
(iii) Endogenous and CAR T-cells in the blood and lesion
(iv) Endogenous T-cells and their phenotypes in the blood
(v) Endogenous and CAR T-cells and their phenotypes in the blood and lesion

**Fig 1:**
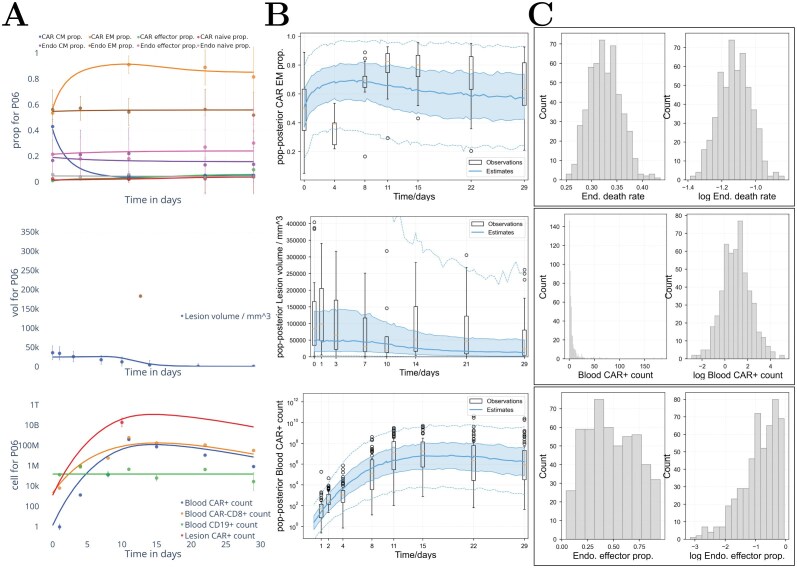
**Example outputs from fitting to the model.** (A) The mean fit of model 5 (see Section 2.1) to patient 06, without uncertainty or parameter variability. Each row shows different observables. Presented from the top row to the bottom row, respectively, are the predicted proportions of CAR and endogenous T-cells in each phenotype, the predicted lesion volume, and predicted cell counts (numbers per 5.3 L of blood; average adult blood volume). Fits to all patients for all models are in the Supplementary Materials. (B) Population fit of model 5 to all patients. A subset of outputs is shown; respectively, by row: the predicted proportion of CAR T-cells that are effectors, the predicted lesion volume, and the predicted blood CAR T-cell count. The box plots are across observations (data), whereas the blue lines are model outputs from 1000 virtual patients. Boxes and blue shaded regions are interquartile ranges, and whiskers and blue dashed lines encompass the 90% percentile. The spread of values comes from both inter-patient variability and uncertainty. Virtual patient parameter variability is obtained from draws from the fitted population distribution, in turn tuned to observed differences in individual parameter fits. Uncertainty comes from random values drawn from error (noise) distributions for each patient, which are deduced from a population distribution fit to data analogously to mechanistic model parameters. (C) Distributions of parameters from the Monolix fit to model 5 for three parameters. The death rate of endogenous and CAR T-cells is Gaussian distributed, the initial condition for blood CAR T-cells is log-normally distributed, and the initial proportion of effector cells that are endogenous has a non-standard distribution. ‘Prop’: proportion. ‘Endo/End’: Endogenous. ‘Vol’: Volume.

The relationship between the five models and the schematic of each dynamic model is presented graphically in Fig. 1. The models are hierarchical; each subsequent model contains all the concepts from previous models.

Models are implemented as ordinary differential equations in Python and Monolix; equations are displayed in the Supplementary Text.

### Model fitting

Model fits were obtained primarily through Monolix. We chose population, error, and correlation models using a combination of biological intuition and statistical recommendations from Monolix. Model details and fit parameters are given in Section A.2 of the Supplementary Text.

### Parameter alteration

We considered how changes to population-level parameter values affect patient outputs. We drew 1000 virtual patients from fitted population parameter distributions and found the resulting model outputs. Then, we modified the parameters of all virtual patients by multiplication or addition of a constant factor, re-ran the model, and compared outputs, as detailed in Section A.3 of the Supplementary Text. Note that repeats with 2000 virtual patients did not induce any changes of consequence, highlighting that 1000 virtual patients has been sufficient to accommodate population variability here.

### Sensitivity analyses

The sensitivity of predictions and model outputs to model parameters was calculated using both local and global methods. High sensitivity of a given output to a given parameter indicates that changes in that parameter strongly influence the output. Each method is summarized below.

#### Global sensitivity analysis with eFAST

The extended Fourier Amplitude Sensitivity Test implemented in the SALib package in Python was run with 1000 runs per parameter and 4 harmonics (akin to repeats). The bounds of the analysis were set to three standard deviations above and below the population value for every population distribution acquired from Monolix. A dummy parameter was included to check for significance. Including the dummy parameter and the 4 harmonics, 188 000 model iterations were taken for model 5.

#### Global sensitivity analysis with random forest

The parameter values and model outputs generated for the eFAST algorithm were also used to train a random forest using the sklearn package in Python; 100 trees were grown in the forest for each model output, and other random forest parameters were left at their default values. The ‘importance indices’ generated by the algorithm for training the forest were taken as sensitivity indices. As the forest was not used for prediction, all model data were used for training.

#### Decision tree analysis

A separate random forest was trained on the optimum parameter sets fitted to the data for each patient, again using 100 trees per model output and using all model data to train the forest. There are two methods by which we could train the random forest with optimum parameter values: either the 64 sets of parameter values fitted to each real individual by Monolix (Section 2.2 above), or 1000 virtual patients drawn from the fitted population models extracted from Monolix (Section A.3, Supplementary Material). Each method yields slightly different results.

#### Correlation analysis

The Spearman rank correlation coefficient was calculated between model outputs and model parameters, using the same 64 or 1000 individuals as in the decision tree analysis.

#### Local sensitivity analysis

Local sensitivity was calculated at the optimum parameter values found by fitting models to data. Optimum parameters were increased or decreased by 1%, and the absolute relative change in each model output was found, averaged across all patients.

### Simplified 2014 Lugano Criteria

To understand how response may be impacted by model parameters, we designed an implementation of the 2014 Lugano Criteria [8] that could be applied to the model. Using the average diameter of all lesions, a virtual patient is classified in terms of,

- a Complete Response (CR) if the sum of product of diameters (SPD) has reduced by at least 50% from baseline and the average longest diameter is less than 1.5 cm,
- a Partial Response if the SPD has reduced by at least 50% from baseline,
- Stable Disease if the SPD has reduced by less than 50% from baseline and the patient is not classified as Progressive Disease (PD),
- PD if the SPD has increased by at least 50% from baseline, the longest diameter is greater than 1.5 cm, and the longest diameter has increased by 0.5 cm from nadir if the lesion diameter is less than 2 cm or increased by 1.0 cm otherwise.

When an estimate for multiple lesions is available, instead of their sum, then

- CR requires that all lesions have a longest diameter less than 1.5 cm,
- a patient has PD if a new lesion appears or if *any* lesion satisfies the PD conditions defined above.

## Results

### Model fits

**Fig 2:**
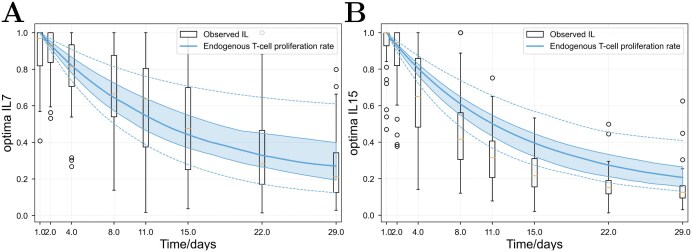
**Plots showing the close relation between the exponential decay of endogenous and CAR T-cell proliferation rates, and the relative (normalized) levels of (A) IL7 and (B) IL15 in patient blood.** Box plots show the IL7 or IL15 data and the blue shaded region shows the interquartile range of predicted proliferation rates (from fits of model 1 to the 64 real patients). The whiskers and blue dashed lines show the 90% percentile.

Fits to both individuals and population parameters were found using Monolix. The fit of model 5 to one individual, the population fit of model 5, and distributions of individual values for several parameters obtained from Monolix are shown in Fig. 2. All individual and population fits for all models are shown in the Supplementary Materials, as are tables for all parameter distributions.

### Interleukin 7 and 15

**Fig 3:**
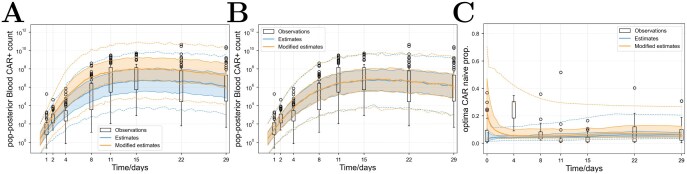
**Plots showing how the distribution of predictions acquired across the virtual patient population for model 5 alters after a change in parameter values.** Box plots show patient data. Blue colours show the median and interquartile range over 1000 virtual patients drawn from population distributions as described in Section A.3 of the Supplementary text. Orange colours indicate the quartiles after changes in parameter values as follows. (A) shows the change in population estimates of the CAR T-cell counts (numbers of cells per 5.3 L of blood) after multiplying the endogenous proliferation rate by 1.5. (B) and (C), respectively, show the change in CAR T-cell counts and the proportion of of naïve CAR T-cells after multiplying the initial proportion of naïve CAR T-cells by 10.

It was found that a model in which the endogenous T-cell proliferation rate falls exponentially over time fits observations best, suggesting that a biological co-factor may correlate with that exponential decay. We found that estimated proliferation rates were highly correlated in time with relative blood concentrations of IL7 (median Spearman RCC across patients of 0.78) and IL15 (0.92). Figure 3 shows the median and interquartile range of their relative concentrations versus the same quartiles of estimated proliferation rates in the patient population, adjusted such that they have the same minimum values. The two curves fit closely, and this holds true for individual patients (see Supplementary Material, Section C, Supplementary Figures, Part 3).

### Alteration of population parameter distributions

**Fig 4:**
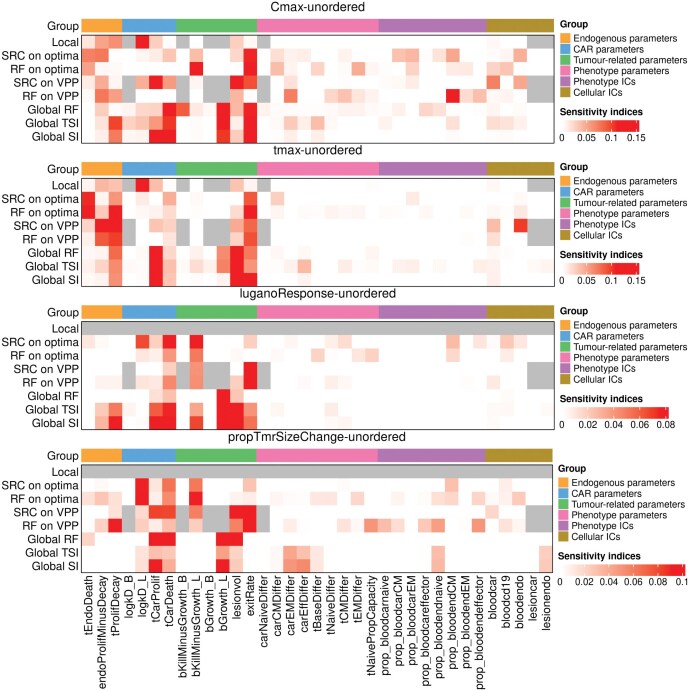
**Heatmap [9] of the normalized sensitivity of various outputs of interest to parameters in model 5, with the apparent sensitivity to a dummy (random) variable subtracted off in all applicable cases.** Darker colours mean greater sensitivity. Different sensitivity techniques give different predictions for which parameters are most important. Group: The parameters on the *x*-axis have been ordered by category, which the coloured horizontal bars indicate. Parameter names: For brevity, short names for model parameters are used. Table A1 in the supplementary materials matches them to descriptive names. Global: a global analysis, across the range of values defined by the population models fit to patient data. SI, TSI: Sensitivity indices and total sensitivity indices obtained from eFAST. RF: Random forest (importance indices). VPP: Random virtual patient population (drawn from fits). SRC: Spearman rank correlation. Optima: the 64 sets of parameter values obtained from fitting to patient data. Local: the change in output after a 1% change in each parameter from the optimum value, averaged over all patients. Cmax: maximum CAR T-cell blood concentration, tmax: time at which Cmax occurs, luganoResponse: the simplified 2014 Lugano criteria (Section 2.5) applied to model outputs. propTmrSizeChange: The relative predicted change in tumour volume from start to end of the simulation. Unordered: As opposed to clustered by their correlation, as shown in the supplementary materials (Fig. A3).

To predict the impact of changes to the CAR T-cell product or patient properties, we altered virtual patients’ parameter values and observed the corresponding change to model outputs. Virtual patients were drawn from the fitted population models for model 5 and used to generate simulated outputs, then virtual patient parameters were modified and simulated again. We analysed the impact of multiplying the initial proportion of naïve CAR T-cells by 10 and the impact of multiplying the endogenous-driven proliferation rate by 1.5, which enhances the proliferation of both endogenous and CAR T-cells. The results are shown in Fig. 4, showing that this model predicts that the change in the naïve proportion has little impact on the number of CAR T-cells in the blood over the first month compared with the change in proliferation rate, and the predicted proportion of CAR T-cells that are naïve approaches the same steady state value regardless of the initial value. Changes to the initial central memory or effector memory proportions have similarly little impact.

### Sensitivity analysis

To find which fitted model parameters are most associated with response, we used various sensitivity analysis techniques. A high sensitivity of a given output to a given parameter indicates that changes in that parameter strongly influence the output. With these analyses, we aim to determine whether lymphodepletion and endogenous-cell-related parameters are most associated with response, or if CAR and antigen-related parameters are. The techniques we used, in order from most to least global are:

- global sensitivity analyses using eFAST and a Random Forest, the parameter bounds for which were defined by the mean ± 3 standard deviations of every patient population distribution,
- a Random Forest analysis and a Spearman Rank Correlation analysis on 1000 random virtual patients drawn from the patient population parameter distributions,
- a Random Forest analysis and a Spearman Rank Correlation analysis on the optimum parameter sets obtained from fitting to 64 individuals,
- a local sensitivity analysis by altering the values of each of the 64 fits by 1%, one parameter at a time.

**Fig 5:**
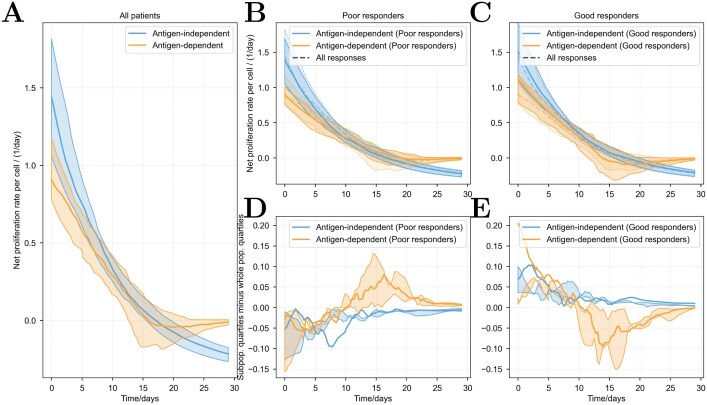
**Estimated proliferation rates of CAR and endogenous T-cells due to antigen-independent (lymphodepletion) and antigen-dependent sources within tumour lesions as a function of time, from fits to the 64 patients.** (A) Quartiles over all patients. The blue curves are the endogenous contribution to proliferation for both endogenous and CAR T-cells, and the orange curves are the antigen-dependent contribution to proliferation. Solid lines are the median over patient fits and the shaded region encompasses the interquartile range. (B) Quartiles over the 32 worst responders. Solid lines are the median over patient fits, the shaded region encompasses the interquartile range, and the dashed lines show the original quartiles over all 64 patients. (C) Quartiles over the 32 best responders. (D, E) The difference between the quartiles of the poor/good responding subpopulations and the quartiles of the entire population. Solid lines are the difference between the medians and the edges of the shaded regions are the difference between the upper and lower quartiles, which are no longer necessarily enveloping the median.

The results of each of these individual analyses are shown in the Supplementary Materials and are summarized in Fig. 5. For each sensitivity analysis, we considered not only the sensitivity of direct model outputs but quantities such as the maximum blood concentration of CAR T-cells (Cmax), the time at which that occurs (tmax), the simplified 2014 Lugano criteria applied to model outputs (Section 2.5) and the proportional change in tumour size (volume estimated from sum of product of diameters). The area under the curve gives nearly identical values to Cmax and is not shown, except in Supplementary Material, Fig. A3. The predicted sensitivity of these quantities to model parameters, shown in Fig. 5, differs both by output and by technique. The parameter axis has been split according to whether parameters are associated with endogenous-driven proliferation, antigen-driven CAR T-cell proliferation, tumour growth and kinetics, phenotypes, or initial cell counts. Recall that CAR T-cells are assumed to be impacted by endogenous-driven dynamics as well as antigen-driven dynamics. In all cases, tumour size and growth rates are predicted to be important. The more local the sensitivity analysis technique, the more that endogenous-related parameters are predicted to be important. The proportional change in tumour size and the response favour antigen-driven proliferation. Endogenous-driven proliferation is slightly more influential on tmax, and Cmax is a mix between endogenous- and antigen-driven proliferation. Phenotype-related parameters and initial cell counts are not predicted to be important for any output by any measure, for the first 30 days.

### Endogenous and CAR contributions to proliferation

Global and local sensitivity analyses yield conflicting conclusions on the relative importance of endogenous-driven proliferation (which affects both endogenous and CAR T-cells) and antigen-driven proliferation on CAR T-cell counts. Fit-agnostic ‘global’ techniques such as eFAST reveal the sensitivity of the model to parameters, whereas drawing virtual patients from the fit populations yields a ‘global’ technique that shows sensitivity of outputs to typical virtual patients, and local modification shows the local sensitivity of the fits to model parameters. To directly determine the relative impacts of each proliferation source, we calculated the proliferation rate of CAR T-cells within the blood and lesion compartments as a function of time, using the parameters obtained from model fits to each patient. In the blood, the number of CD19^+^ cells is constant at the limit of quantitation and (thus) is assumed not to interact with CAR T-cells in the model, so endogenous-driven proliferation dominates there. Proliferation in the lesion is shown in Fig. 6. The rate of endogenous-driven proliferation is found to be higher than antigen-driven proliferation, but not by more than two-fold. We ranked patients by their response in a tiered approach. Patients were first ordered by their month 1 response, then patients with similar outcomes at month 1 were ordered by their month 3 response, and then by their month 6 response, as assessed in the TRANSCEND NHL-001 study [1]. We split this list of patients into two equal-sized groups, labelled as ‘good’ and ‘poor’ responders in the figure. This shows that both endogenous-driven and antigen-driven proliferation are higher in good responders than poor responders. In good responders, antigen-driven response is higher than the population average at early times, and in poor responders, it is higher than the population average at around day 15.

## Discussion

In this study, we produced models of CAR and endogenous T-cell dynamics in anti-lymphoma responses. Models were constructed hierarchically, so that smaller models could be fit sequentially. This procedure aided fitting, as not all parameters were considered simultaneously, but it imposed strong assumptions on model structure. The structure of model 1 is retained in all five models, so endogenous T-cell proliferation slows over time and is unaffected by CAR T-cells and the ongoing response to the lymphoma lesion. This assumption fits the data well but may not be strictly true. It has been observed that endogenous T-cells can contribute toward an antigen-tumour response long after CAR T-cell therapies [10]. This effect was neglected in this work because the initial number of anti-tumour endogenous T-cells is expected to be low; a patient has lymphoma indicates that their current repertoire of T-cells does not produce a sufficient number of anti-tumour T-cell receptors, which would be further impacted by lymphodepletion. Another consequence of simulated T-cell proliferation falling over time is that the number of endogenous T-cells is predicted to fall at late times, but in reality, homeostasis should maintain this pool, and it is possible that neoantigens generated by the CAR T-cell response may stimulate new populations of T-cells. In this study, we focus on the first 30 days posttherapy, so we expect the endogenous anti-tumour effect to remain small and the progression into T-cell homeostasis is not observed.

The hierarchical model structure means that the total endogenous cell count predicted by all models is the same. To achieve this, the proliferation rate for all four endogenous phenotypes in model 4 is assumed to be identical, which is counter intuitive. This is well-supported by the data, as no phenotype proportion ever reaches zero or one in any patient. If one of the phenotypes had a higher proliferation rate than the others, then that proportion would quickly approach 100% in the absence of negative feedback mechanisms (e.g. maintaining a naïve pool of cells). We were not able to find a set of equations with independent proliferation rates that matched the data for all phenotypes. One possible explanation is that the likely greatest source of proliferation for the endogenous T-cells in the first 30 days, lymphodepletion, could affect T-cells independently of their phenotype.

The model predicts that the proportions of CAR T-cells in each phenotype inevitably approach equilibrium, and changes to initial conditions have little predicted impact on results (Fig. 4). This is partially due to the data; the proportion of each phenotype in most patients remains relatively static for prolonged periods. These two model features—that total cell counts are independent of phenotype proportions and that phenotype proportions quickly reach equilibrium—mean that changing phenotype proportions is only predicted to impact CD19^+^ cell killing, and not overall CAR T-cell counts. This is surprising: we expected to see phenotype proportions be important for CAR T-cell dynamics and patient outcomes. For example, Fraietta *et al.* compared the gene expression profiles of CD19-directed T-cell therapies administered in the clinic and observed that increased expression of naïve or early memory cells correlated with patient response [5]. An alternative model, which relaxes the requirement that the sum of all phenotype counts is independent of the proportions of cells in each phenotype, but is still able to fit each proportion, might produce this behaviour. Such a model would not be able to assume that cells of each phenotype proliferate exponentially with different rates without introducing an exponentially increasing ratio between two or more phenotypes. However, regulation of T-cell proliferation by a different factor such as cytokines instead of exponential growth might reproduce the steady state of phenotype proportions without nullifying their effect on dynamics.

The estimated T-cell proliferation rates were found to be time-correlated with patient IL7 and IL15 blood concentrations (Fig. 3). IL-7 and IL-15 are known to be related to T-cell proliferation and homeostasis [11, 12], so this observation could be related to lymphodepletion; however, correlation analysis (heatmap in Supplementary Materials, Section C, Supplementary Figures, Section 5, part 1) indicates that proliferation rates are also correlated with MCP-1, MIP-1β, and the proportion of T-cells that are naïve or are expressing CCR4. This suggests that the correlations may instead be a general marker of immune activity, or as we used the Spearman rank correlation, it may simply be that all these quantities change monotonically over the first 30 days with no causal relationship between them. However, previous studies have found an association between these cytokines and response. Kochenderfer *et al.* found that higher serum levels of IL15 induced by low-dose chemotherapy were associated with higher peak CAR T-cell levels and remission in various lymphomas [13], and Hirayama *et al.* found that more intensive lymphodepletion was associated with higher postlymphodepletion levels of MCP-1 and IL-7, which in turn were associated with better progression free survival in non-Hodgkin lymphoma patients [14]. Both observations are consistent with our finding, but we go further by specifically correlating endogenous and CAR T-cell proliferation with levels of IL-7 and IL-15. As discussed above, incorporating such cytokines directly into the differential equations may be required to more accurately model how each T-cell phenotype impacts T-cell dynamics.

For Non-Hodgkin Lymphoma and Diffuse Large B-Cell Lymphoma, the bulk of tumour cells exist as solid or diffuse lesions, from which obtaining biopsies is non-trivial and thus lesion data are sparser than blood data for most patients. In this context, a population level approach is ideal for constraining model predictions for those individuals in which there is no data and for better predicting population-level changes. To further reduce parameter unidentifiability or uncertainty, we assume that,

- the proportion of cells in each phenotype in the lesion is equal to those in the blood,
- all dynamic parameters, except the killing, dissociation, and B-cell growth rates, are the same in blood and lesion,
- activity outside the blood and tumour can be ignored; as peripheral data were not available, we used a two-compartment model,
- tumour elimination happens evenly in all lesions, so that total lesion volume can be used,
- tumour cells have identical volumes and lesion shapes are sufficiently regular that the number of malignant cells can be estimated from the product of lesion diameters,
- the ratio of CAR T-cells in the blood to the tumour is always constant (as expected if extravasation is faster than proliferation, killing or death),
- Lactate Dehydrogenase concentration can be used to interpolate tumour burden at intermediate times, from the products of lesion diameters measured at the study start and end,
- T-cells do not contribute to lesion volumes, to avoid uncertainty in the extent of T-cell infiltration and whether lesion or lymph node *organ* volume should be used as the T–B-cell interaction volume.

Each assumption introduces uncertainty from modelling errors, particularly in lesion volumes, but was necessary for model fitting. We do not explore the role of the tumour composition or microenvironment in the model, because we could not quantify it. Similarly, the serum concentration of CD19^+^ cells for most patients is always near the limit of quantification, and so their dynamics and interactions with CAR T-cells could not be quantified. This results in a model that predicts that the CAR T-cells’ dynamics are primarily driven by themselves, rather than the patient’s tumour burden.

Previous studies have reported that CAR T-cell longevity is important for durable patient response. We focus only on the first 30 days: the initial expansion and decline phases, which may explain why we do not find an association between memory T-cell phenotypes and response. Authors such as Stein *et al.* predict a two-phase decline [2], but the second, slower decline phase occurs after several months. In contrast, our model would predict that the first decline phase would continue indefinitely, with endogenous and CAR T-cell numbers falling to zero. Without data beyond the first 30 days, it is impossible to parameterize the second, slower decline, but this is a trivial model extension with appropriate data. A conclusion of this study (and others) is that early expansion of the CAR T-cells is a critical factor for efficacy, to ensure that there are enough CAR T-cells to overwhelm the target cells. This is a necessary but insufficient condition for response in many patients; persistence is important but irrelevant without sufficient initial expansion.

We used several techniques to assess the sensitivity of model outputs to parameters, ranging from global to local sensitivity analyses, which differ in their predictions of which parameters are most important, with more local techniques predicting that endogenous-related parameters are more important. Our global analyses used parameter ranges defined by population parameter distributions. Often, the spread from log-normal distributions is much larger than for normally distributed parameters, suggesting that the predicted importance of log-normally distributed parameters such as antigen-driven proliferation was overestimated. While more global sensitivity techniques explore the general behaviour of the model, local techniques may be more relevant in this application, as they focus on the area of parameter space most relevant to patient data from the NHL-001 clinical trial. This would suggest that parameters that can be related to proliferation of both endogenous and CAR T-cells are most relevant for understanding the first 30 days of the response of patients in the trial, but this again may be due to structure of the model as discussed above. Further discussion of the interpretation of sensitivity techniques can be found in the literature [15–17].

To avoid the inconsistent conclusions from sensitivity analyses, we determined the contributions of endogenous-driven and antigen-driven proliferation to CAR T-cell dynamics directly, in Section 3.5. Endogenous-driven proliferation was found to be 50% faster than antigen-driven, which exponentially impacts CAR T-cell counts. This leads to the conclusion that lymphodepletion, the hypothesized mechanism behind the ‘endogenous-driven’ proliferation, is critical for early CAR T-cell responses. The largest numerical change between responders and non-responders is that responders had stronger early antigen-driven proliferation, despite the absolute values being less than for endogenous-driven proliferation. The results of this and the sensitivity analyses together suggest that both antigen and endogenous-driven proliferation are important, but since the endogenous and CAR T-cells are so closely related, if it is difficult to observe the total count of CAR T-cells, it might suffice to track the count of *endogenous* T-cells to determine whether a patient is responding poorly. If the endogenous T-cells are not expanding, it may be likely that the CAR T-cells are not either.

## Conclusion

In this study, we sought to determine which factors most strongly influence (the lack of) patient response in the first 30 days postinfusion of CAR T-cells, in the context of the anti-CD19 product, liso-cel. Fitting the model to data leads to the finding that simulated CAR T-cell counts are strongly influenced by an antigen-independent source of proliferation common to both endogenous and CAR T-cells. We found that the parameters describing antigen-independent proliferation in the model were highly correlated with relative patient concentrations of IL-7 and IL-15, suggesting a link to lymphodepletion. The link between CAR and endogenous T-cell proliferation implies a useful surrogate in the first month of response: where endogenous T-cells are not expanding, CAR T-cells may be likely not to, either. It was found that the impact of the proportions of CAR T-cells in different (naïve, memory, effector) phenotypes on the total CAR T-cell count or on response was modest, compared with effects associated with lymphodepletion, suggesting that it is at only later times that memory CAR T-cells become important for maintenance of response, as other studies have found.

In future, this framework is intended for use to investigate strategies that could evaluate and improve patient responses to a modified CAR-T product in the first 30 days.

## Study highlights

### What is the current knowledge on the topic?

The dose–response relationship for CAR T-cells is relatively weak because cells transferred into a patient immediately proliferate. The effective dose depends on many patient-specific factors, from tumour burden to cytokine environment.

### What question did this study address?

Identifying the strongest determinants of patient response in the first 30 days after anti-CD19 CAR T-cell transfusion, and how changes to controllable factors might affect dynamics and response in that timescale, by fitting models to patient data.

### What does this study add to our knowledge?

Antigen-dependent and independent sources of proliferation affect CAR T-cell dynamics, antigen-independent proliferation was highly correlated with patient IL-15 and IL-7 blood concentrations, and the proportions of naïve/memory/effector T-cells were found to have limited impact in the first 30 days.

### How might this change drug discovery, development, and/or therapeutics?

We show how models can link endogenous and CAR T-cell dynamics, which may be useful to predict poor responders by observing endogenous T-cell dynamics. Our framework can be utilized for other constructs and indications, to test product alterations or biological hypotheses.

## Data Availability

The clinical data associated with this study are proprietary and not publicly available. The results drawn from this study are summarized in the Supplementary Methods, Section C, Supplementary Figures, where a DOI link is provided.

## Supplementary information titles

- Supplementary methods
  Definition of models
  Fitting in Monolix
  Fitting in Python
  Extrapolation of results
  Correlation analysis
  Flow cytometry
  Definition of data
- Supplementary results
  Logistic growth
  Extrapolation of fits
  Clustered heat maps
- Supplementary figures
- Supplementary tables

## Rights Retention Statement

For the purpose of Open Access, a CCBY public copyright licence is applied to any Author Accepted Manuscript version arising from this submission.

## Permission to Reproduce

This is not applicable; e.g. no figures have been used from other publications.

## References

[1] Abramson JS , Palomba ML, Gordon LI et al Lisocabtagene maraleucel for patients with relapsed or refractory large B-cell lymphomas (TRANSCEND NHL 001): a multicentre seamless design study. Lancet 2020; 396(10254):839–52. ISSN 0140-6736. doi: 10.1016/S0140-6736(20)31366-0. 32888407

[2] Stein AM , Grupp SA, Levine JE et al Tisagenlecleucel model-based cellular kinetic analysis of chimeric antigen receptor–T cells. CPT: Pharmacometrics Syst Pharmacol 2019; 8(5):285–95. ISSN 2163-8306. doi: 10.1002/psp4.12388. 30848084 PMC6539725

[3] Singh AP , Zheng X, Lin-Schmidt X et al Development of a quantitative relationship between CAR-affinity, antigen abundance, tumor cell depletion and CAR-T cell expansion using a multiscale systems PK-PD model. mAbs 2020;12(1):1688616. ISSN 1942-0862. doi: 10.1080/19420862.2019.1688616. 31852337 PMC6927769

[4] Singh AP , Chen W, Zheng X et al Bench-to-bedside translation of chimeric antigen receptor (CAR) T cells using a multiscale systems pharmacokinetic-pharmacodynamic model: a case study with anti-BCMA CAR-T. CPT: Pharmacometrics Syst Pharmacol 2021;10(4): 362–76. ISSN 2163-8306. doi: 10.1002/psp4.12598. 33565700 PMC8099446

[5] Fraietta JA , Lacey SF, Orlando EJ et al Determinants of response and resistance to CD19 chimeric antigen receptor (CAR) T cell therapy of chronic lymphocytic leukemia. Nat Med 2018;24:563–71. ISSN 1546-170X. doi: 10.1038/s41591-018-0010-1. 29713085 PMC6117613

[6] Saini N , Neelapu SS. CAR Treg cells: prime suspects in therapeutic resistance. Nat Med 2022;28:1755–6. ISSN 1546-170X. doi: 10.1038/s41591-022-01998-7. 36109644 PMC12036812

[7] Good Z , Spiegel JY, Sahaf B et al Post-infusion CAR TReg cells identify patients resistant to CD19-CAR therapy. Nat Med 2022;28:1860–71. ISSN 1546-170X. doi: 10.1038/s41591-022-01960-7. 36097223 PMC10917089

[8] Cheson BD , Fisher RI, Barrington SF et al; Alliance, Australasian Leukaemia and Lymphoma Group, Eastern Cooperative Oncology Group, European Mantle Cell Lymphoma Consortium, Italian Lymphoma Foundation, European Organisation for Research, Treatment of Cancer/Dutch Hemato-Oncology Group, Grupo Español de Médula Ósea, German High-Grade Lymphoma Study Group, German Hodgkin’s Study Group, Japanese Lymphorra Study Group, Lymphoma Study Association, NCIC Clinical Trials Group, Nordic Lymphoma Study Group, Southwest Oncology Group, and United Kingdom National Cancer Research Institute. Recommendations for initial evaluation, staging, and response assessment of Hodgkin and non-Hodgkin lymphoma: the Lugano classification. J Clin Oncol 2014;32(27):3059–68. ISSN 1527-7755. doi: 10.1200/JCO.2013.54.8800. 25113753 PMC4979083

[9] Gu Z. Complex heatmap visualization. iMeta 2022;1(3):e43. ISSN 2770-596X. doi: 10.1002/imt2.43. 38868715 PMC10989952

[10] Reiss DJ , Do T, Kuo D et al Multiplexed immunofluorescence (IF) analysis and gene expression profiling of biopsies from patients with relapsed/refractory (R/R) diffuse large B cell lymphoma (DLBCL) treated with lisocabtagene maraleucel (liso-cel) in transcend NHL 001 reveal patterns of immune infiltration associated with durable response. Blood 2019;134:202. ISSN 0006-4971. doi: 10.1182/blood-2019-127683.

[11] Tan JT , Ernst B, Kieper WC et al Interleukin (IL)-15 and IL-7 jointly regulate homeostatic proliferation of memory phenotype CD8+ cells but are not required for memory phenotype CD4+ cells. J Exp Med 2002;195(12): 1523. doi: 10.1084/jem.20020066. 12070280 PMC2193564

[12] Prlic M , Lefrancois L, Jameson SC. Multiple choices: regulation of memory CD8 T cell generation and homeostasis by interleukin (IL)-7 and IL-15. J Exp Med 2002;195(12):f49. doi: 10.1084/jem.20020767. 12070294 PMC2193558

[13] Kochenderfer JN , Somerville RPT, Lu T et al Lymphoma remissions caused by anti-CD19 chimeric antigen receptor T cells are associated with high serum interleukin-15 levels. J Clin Oncol 2017;35(16):1803. doi: 10.1200/JCO.2016.71.3024. 28291388 PMC5455597

[14] Hirayama AV , Gauthier J, Hay KA et al The response to lymphodepletion impacts PFS in patients with aggressive non-Hodgkin lymphoma treated with CD19 CAR T cells. Blood 2019;133(17):1876. doi: 10.1182/blood-2018-11-887067. 30782611 PMC6484391

[15] Marino S , Hogue IB, Ray CJ, Kirschner DE. A methodology for performing global uncertainty and sensitivity analysis in systems biology. J Theor Biol 2008;254(1):178–96. ISSN 0022-5193. doi: 10.1016/j.jtbi.2008.04.011. 18572196 PMC2570191

[16] Wentworth MT , Smith RC, Banks HT. Parameter selection and verification techniques based on global sensitivity analysis illustrated for an HIV model. IAM-ASA J Uncertain Quantif 2016;4(1):266–97.

[17] Cheng Y , Straube R, Alnaif AE et al Virtual populations for quantitative systems pharmacology models. Methods Mol Biol 2022;2486:129–79. ISSN 1940-6029. doi: 10.1007/978-1-0716-2265-0_8. 35437722

