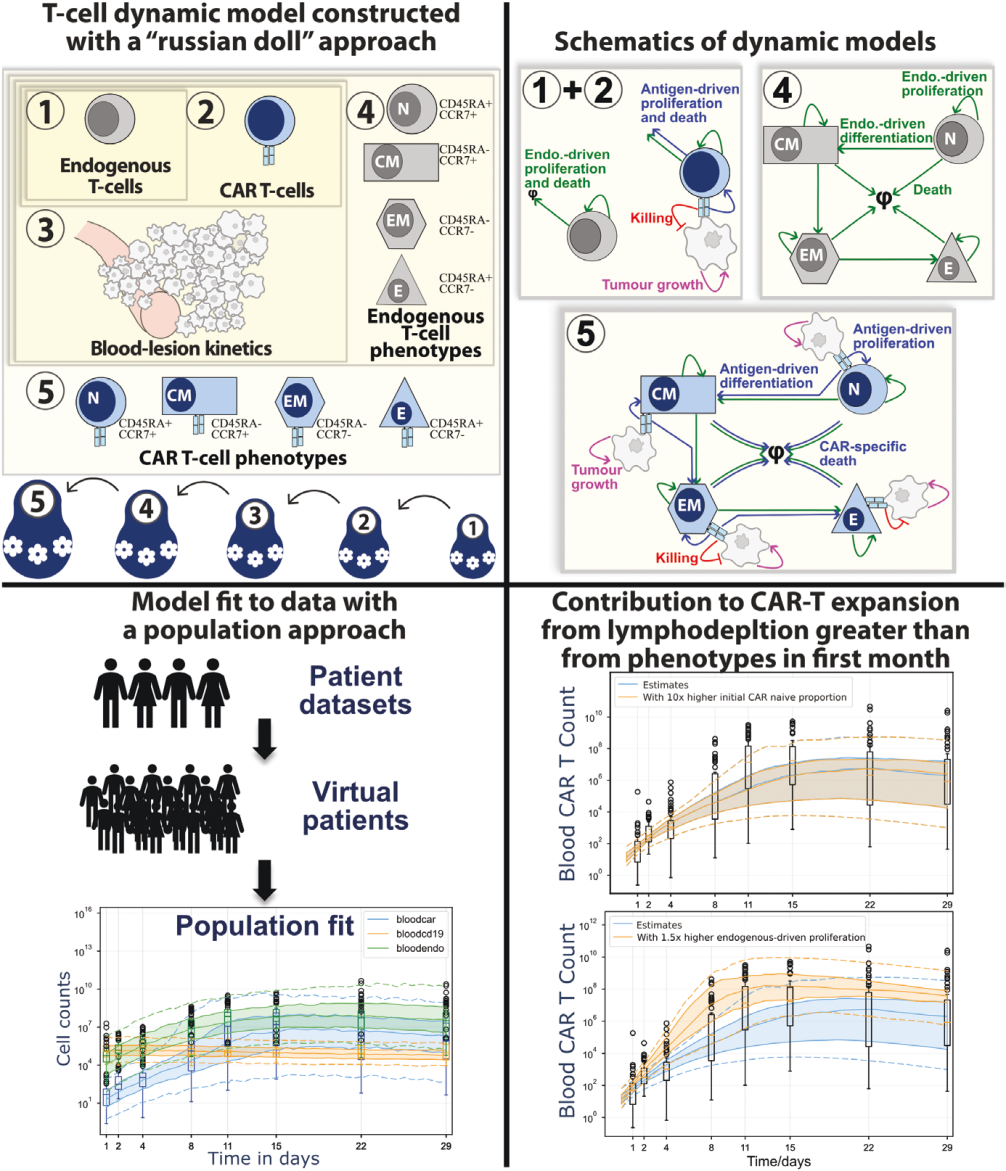# Correction to: Applying population mechanistic modelling to find determinants of chimeric antigen receptor T-cells dynamics in month-one lymphoma patients

**DOI:** 10.1093/immadv/ltaf028

**Published:** 2025-09-10

This is a correction to: Liam V Brown, Mark McConnell, Robert Rosler, Leanne Peiser, Brian J Schmidt, Alexander V Ratushny, Eamonn A Gaffney, Mark C Coles, Applying population mechanistic modelling to find determinants of chimeric antigen receptor T-cells dynamics in month-one lymphoma patients, *Immunotherapy Advances*, Volume 5, Issue 1, 2025, https://doi.org/10.1093/immadv/ltaf001

The graphical abstract is emended from the original publication. The difference is the inclusion of the top-right cell ‘Schematics of dynamic models’.